# Evaluating the Efficacy of the *Drinks*:Ration Mobile App to Reduce Alcohol Consumption in a Help-Seeking Military Veteran Population: Randomized Controlled Trial

**DOI:** 10.2196/38991

**Published:** 2022-06-20

**Authors:** Daniel Leightley, Charlotte Williamson, Roberto J Rona, Ewan Carr, James Shearer, Jordan P Davis, Amos Simms, Nicola T Fear, Laura Goodwin, Dominic Murphy

**Affiliations:** 1 Institute of Psychiatry, Psychology & Neuroscience King’s Centre for Military Health Research King's College London London United Kingdom; 2 Department of Biostatistics and Health Informatics King's College London London United Kingdom; 3 King’s Health Economics King's College London London United Kingdom; 4 Suzanne Dworak-Peck School of Social Work University of Southern California Los Angeles, CA United States; 5 Academic Department of Military Mental Health King's College London London United Kingdom; 6 British Army London United Kingdom; 7 Division of Health Research Lancaster University Lancaster United Kingdom; 8 Combat Stress Leatherhead United Kingdom

**Keywords:** military, veteran, digital health, alcohol misuse, smartphone, mobile health, mHealth, alcohol intervention, digital intervention, mental health, smartphone application, health intervention, alcohol consumption

## Abstract

**Background:**

Alcohol misuse is higher in the UK armed forces (AF) than in the general population. Research demonstrates that alcohol misuse persists after an individual leaves service, and this is notably the case for those who are seeking help for a mental health difficulty. Despite this, there is no work on testing a mobile alcohol reduction intervention that is personalized to support the UK AF.

**Objective:**

To address this gap, we investigated the efficacy of a 28-day brief alcohol intervention delivered via a mobile app in reducing weekly self-reported alcohol consumption among UK veterans seeking help for mental health difficulties.

**Methods:**

We performed a 2-arm participant-blinded randomized controlled trial (RCT). We compared a mobile app that included interactive features designed to enhance participants’ motivation and personalized messaging (intervention arm) with a version that provided government guidance on alcohol consumption only (control arm). Adults were eligible if they had served in the UK AF, were currently receiving or had received clinical support for mental health symptoms, and consumed 14 units (approximately 112 g of ethanol) or more of alcohol per week. Participants received the intervention or the control mobile app (1:1 ratio). The primary outcome was a change in self-reported weekly alcohol consumption between baseline and day 84 assessed using the validated Timeline Follow Back for Alcohol Consumption (TLFB) (prior 7 days), with a secondary outcome exploring self-reported change in the Alcohol Use Disorder Identification Test (AUDIT) score.

**Results:**

Between October 2020 and April 2021, 2708 individuals were invited to take part, of which 2531 (93.5%) did not respond, 54 (2%) were ineligible, and 123 (4.5%) responded and were randomly allocated (62, 50.4%, intervention; 61, 49.6%, control). At day 84, 41 (66.1%) participants in the intervention arm and 37 (60.7%) in the control arm completed the primary outcome assessment. Between baseline and day 84, weekly alcohol consumption reduced by –10.5 (95% CI –19.5 to –1.5) units in the control arm and –28.2 (95% CI –36.9 to –19.5) units in the intervention arm (*P*=.003, Cohen *d*=0.35). We also found a significant reduction in the AUDIT score of –3.9 (95% CI –6.2 to –1.6) in the intervention arm (Cohen *d*=0.48). Our primary and secondary effects did not persist over the longer term (day 168). Two adverse events were detected during the trial.

**Conclusions:**

This study examined the efficacy of a fully automated 28-day brief alcohol intervention delivered via a mobile app in a help-seeking sample of UK veterans with hazardous alcohol consumption. We found that participants receiving *Drinks*:Ration reduced their alcohol consumption more than participants receiving guidance only (at day 84). In the short term, we found *Drinks*:Ration is efficacious in reducing alcohol consumption in help-seeking veterans.

**Trial Registration:**

ClinicalTrials.gov NCT04494594; https://tinyurl.com/34em6n9f

**International Registered Report Identifier (IRRID):**

RR2-10.2196/19720

## Introduction

Evidence has shown alcohol misuse is substantially higher in the United Kingdom’s armed forces (AF) than the UK general population [1,2]. Research has demonstrated that alcohol misuse persists after leaving service [3], and 43% of veterans seeking treatment for a mental health difficulty report misusing alcohol [4]. In the United Kingdom, a veteran is defined as an individual serving at least 1 day of paid employment in the AF. Alcohol misuse often co-occurs with posttraumatic stress disorder (PTSD), anxiety, or depression and frequently used as a coping mechanism [5].

Research has shown that help-seeking veterans (those seeking support in a clinical setting) misusing alcohol attend fewer mental health appointments and are more likely to have a negative perception of mental health treatment [6]. This is, in part, due to those misusing alcohol being denied access to mental health treatment services until they have reduced the hazardous drinking. Thus, interventions that target drinking behavior need to be developed as this may enhance engagement with mental health services and improve mental health outcomes and quality of life.

The past 5 years have seen a growing treatment gap, with patients waiting longer for mental health referrals and treatment in the United Kingdom. This has further been exacerbated by the COVID-19 pandemic. To address these issues, modes of intervention delivery have shifted from in-person to web-based to mobile-based delivery [7]. Mobile interventions for alcohol misuse in the United Kingdom, such as Drink Less [8] and Drinkaware [9], have several advantages over web-based delivery, including (1) more holistic delivery of behavior changes, (2) the use of mobile sensors and wearables to inform decision-making, (3) avoiding the stigma associated with receiving help in person, and (4) convenience since they can be used when the individual prefers (discretely or openly). Mobile interventions also offer a more cost-efficient way to deliver behavior change techniques (BCTs, the specific and active component of an intervention designed to change behavior [10]) for reducing alcohol use.

Existing alcohol apps targeted at the general public include self-monitoring apps (eg, Drink Less [8], Drinkaware [9], One You Drinks Tracker [11]), where users are encouraged to regularly record and monitor (via visualizations) their alcohol consumption. Self-monitoring has been found to be associated with improved outcomes and is an effective BCT for reducing alcohol use. A recent review of personalized digital interventions found reductions in hazardous and harmful alcohol consumption to be associated with behavior substitution, problem solving, and providing a credible source of information [12]. Another review also identified the role that personalized notifications play in promoting positive changes in behavior [13]. However, current mobile interventions focused on the general population do not target aspects experienced by the AF community, such as individual beliefs, prevailing social context, comorbid mental health problems, military service experience, and perceptions of consumption [14]. Further, existing apps do not cater for the excessive amounts of alcohol consumed by UK AF personnel.

To date, there is no published work that seeks to test a brief automated mobile intervention alcohol reduction app that is personalized to support UK AF, considering their military experiences. To address this, we developed the *Drinks*:Ration app (previously called Information about Drinking for Ex-Serving personnel [InDEx]; see [15-18]) to support UK AF veterans to reduce the amount they drink.

We conducted a randomized controlled trial (RCT) to assess the efficacy of a 28-day alcohol intervention delivered via *Drinks*:Ration in reducing self-reported weekly alcohol consumption by day 84 follow-up among veterans who drink at hazardous or harmful levels (ie, drinking at a level likely to cause harm) and are currently receiving, or have previously received, support for mental health symptoms in a clinical setting.

## Methods

### Study Design and Hypotheses

This was a 2-arm participant-blinded (single-blinded) RCT (1:1) to compare a mobile app that provided government guidance on alcohol consumption only (control arm) with the mobile app *Drinks*:Ration, a personalized app based on BCT principles. We hypothesized that the intervention arm would be efficacious in reducing alcohol consumption when compared to the control arm.

Both the control and intervention arms were delivered via the *Drinks*:Ration app. Participants in the control arm were given access only to the alcohol consumption guidance and a unit calculator based on guidance issued by the UK Chief Medical Officer. Those in the intervention arm were given access to the full version of the app, which included theoretically driven components and personalized messaging. This included individualized normative guidance alongside features designed to enhance participants’ motivation through interactive guidance focused on self-efficacy to help modify alcohol consumption. Participants in both arms were asked to use the app for a minimum of 28 days. After this, they could continue to use the app, but they did not receive personalized messaging. This was undertaken to assess the long-term effectiveness of the app.

This study was designed such that the control arm structurally resembled the intervention arm but excluded active intervention techniques, such as a drinks diary, drinks in pixels, and drinking zones (based on the Global Positioning System [GPS] location). This approach increased uniformity across the arms (eg, ensuring both arms received a digital intervention) and maintained treatment allocation concealment.

Data were collected at baseline and follow-up assessments at 84 and 168 days postbaseline. Additional questionnaires were collected on days 7, 14, and 21. This information was used to personalize the *Drinks*:Ration app for the intervention arm only. Please refer to the published trial protocol for further details [19].

### Ethical Considerations

This trial was approved by the local ethics committee of King’s College London (HR-19/20-17438).

### Procedure and Participants

Participants were recruited between October 2020 and April 2021 in succession via a clinical group, an existing research cohort [2], and social media [20]. The clinical group was derived from Combat Stress, a third-sector charitable organization that provides mental health services, including substance misuse management, to UK veterans. The research cohort was the King’s Centre for Military Health Research health and well-being longitudinal cohort study [2], where a sample of self-reported help seekers were identified and extracted. Social media platforms, such as Facebook and Twitter, were also used to promote the RCT via free and paid promotional advertisements with a link enabling potential users to express an interest in taking part (for further information, please see [20]).

Potential participants were invited to take part via email with an explanation of the study, a link to the participant information sheet, and instructions on how to download *Drinks*:Ration using a unique quick response (QR) code. Once participants had downloaded the app, they were asked to report alcohol consumption using the validated Timeline Follow Back for Alcohol Consumption (TLFB) [21] for the prior 7 days and confirm their military serving status. Those meeting the study eligibility criteria were allowed to proceed and complete the baseline questionnaire.

Eligibility was assessed at baseline. To be included in this RCT, participants needed to download the *Drinks*:Ration app onto an iOS or Android device; be aged 18 years or older; currently reside in the United Kingdom; consume at least 14 UK units (approximately 112 g of ethanol) of alcohol or more per week at baseline (hazardous or harmful levels of alcohol consumption); confirm that they currently receive, or had received, support for mental health symptoms in a clinical setting; provide a mobile phone number; and be a veteran of the UK AF.

### Sample Size

A power calculation was performed based on Alcohol Use Disorder Identification Test (AUDIT) data previously reported from Combat Stress [5]. To detect a difference in alcohol consumption of 4 UK units (approximately 40 g of alcohol per week) between the control and intervention arms at day 84, with a 2-sided 5% significance level and a power of 80%, we needed a sample of 37 participants per arm with complete primary outcome assessments. We selected 4 UK units based on reductions observed in similar studies [8,22,23] and reductions observed in the feasibility trial of *Drinks*:Ration, which found a 7-unit decrease at week 4. To allow for attrition of 40%, we aimed to recruit a total of 124 participants (62, 50%, per arm). To account for an expected response rate of 30%, we estimated that we would need to invite 620 veterans to participate in the study.

### Randomization and Masking

Randomization was carried out automatically as part of the *Drinks*:Ration platform. When participants registered for the app, they were assigned a unique identifier and asked to provide their gender. They were then randomly allocated [1:1] to receive either the control or the intervention arm. Stratification was used to ensure equal gender distribution across arms. This is because those who identify as female only represent approximately 10% of the UK AF.

The randomization procedure was based on a list of random numbers computer-generated by the *Drinks*:Ration platform. All members of the research team were blind to participant treatment allocations except for authors DL and CW. CW conducted participant management, and DL led the development of the *Drinks*:Ration app, had access to raw study data, and conducted the primary analyses. Except for automated weekly backups, access to the data was disabled.

Participants were not informed of their treatment allocation. However, they may have been able to deduce their allocation condition based on app content. As the intervention was automated and delivered via an app, there was no contact between researchers and participants during the intervention unless a risk to health (adverse event) had been detected or if technical problems arose [19]. An adverse event in this study was defined as participants reporting (via the drinks diary or during contact with the research team) that they had consumed more than 25 UK units of alcohol within a 24-hour period. Once detected, a clinician would contact the participant over the telephone to check their health and provide signposting to other services (which are listed in the app). The clinician would not disclosure the treatment arm allocation.

### Intervention

**Figure 1:**
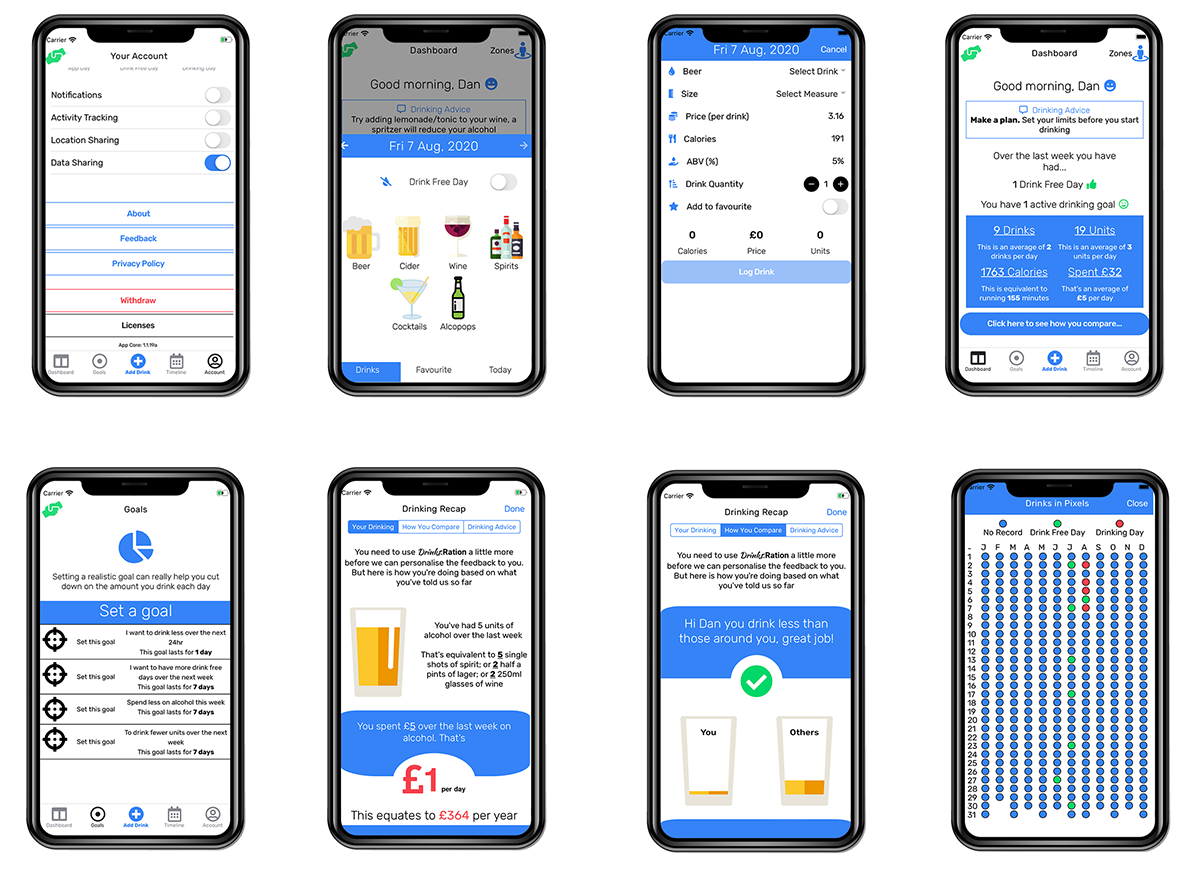
Example screenshots extracted from the *Drinks*:Ration app.

*Drinks*:Ration (formerly called InDEx [15-17]) was developed by the King’s Centre for Military Health Research (at King’s College London) and the University of Liverpool following the Medical Research Council Complex Intervention Guidelines using co-design methodology in collaboration with end users. The app was designed to support veterans drinking at hazardous or harmful levels by providing bespoke advice and support over a minimum of 28 days. The app was developed without any organizational branding to promote use. The iterative development process, theoretical framework, and feasibility trial are reported elsewhere [15-17,19]. Briefly, *Drinks*:Ration was developed and tested with 5 core modules (see Figure 1):

- Account management: Participants can modify personal information (eg, first name and mobile number) and app parameters (eg, automatic logout, clear local storage, data sharing permission, and leaving the study).
- Questionnaires and individualized normative guidance: This captures the participant’s response to a set of questions and aggregates responses to produce an individualized infographic representing the participant’s alcohol consumption in comparison to the general population, the AF community, and other participants of the *Drinks*:Ration app.
- Self-monitoring and guidance: This records alcohol consumption by participants and provides a range of visualizations to allow consumption monitoring. Further, participants can customize the visualizations with metrics they find relevant (eg, calories, cost, or exercise required).
- Goals (setting and review): Participants can set goals based on the implementation intentions (if and then) methodology; visualizations provide guidance on progress toward achieving specified goals.
- Personalized messaging: Participants are sent tailored messages via push notifications or short message service (SMS) text messages that provide prompts to use the drinks diary, suggest alternative behaviors, and provide guidance on goals (see Multimedia Appendix 1 for example messaging).

Participants in the intervention arm completed additional questionnaires on their mood and general mental health each week. These responses were used to personalize app content, push notifications, and SMS text messages.

### Measures

A summary of measures and data collection schedule is provided in Table 1 (and see [19]). Measures were the same for the intervention and control arms.

**Table 1:** Summary of measures and data collection timepoints. Days 7, 14, and 21 measures were used to personalize the *Drinks*:Ration app and apply to the intervention arm only.

Measure	Day 0 (baseline)	Day 7	Day 14	Day 21	Day 28	Day 84 (primary endpoint)	Day 168	
Informed consent	I^a^/C^b^	N/A^c^	N/A	N/A	N/A	N/A	N/A	
Sociodemographics	I/C	N/A	N/A	N/A	N/A	N/A	N/A	
Depression (2-item Patient Health Questionnaire [PHQ2]) [24]	I/C	I/C	I/C	I/C	I/C	I/C	I/C	
Anxiety (2-item Generalized Anxiety Disorder [GAD2]) [25]	I/C	I/C	I/C	I/C	I/C	I/C	I/C	
International Trauma Questionnaire (ITQ) for PTSD^d^ [26]	I/C	N/A	N/A	N/A	I/C	I/C	I/C	
AUDIT^e^ [27]	I/C	N/A	N/A	N/A	I/C	I/C	I/C	
7-day TLFB^f^ [21]	I/C	N/A	N/A	N/A	I/C	I/C	I/C	
Usability evaluation (MAUQ^g^) [28]	N/A	N/A	N/A	N/A	I/C	N/A	N/A	

^a^I: intervention arm.

^b^C: control arm.

^c^N/A: not applicable.

^d^PTSD: posttraumatic stress disorder.

^e^AUDIT: Alcohol Use Disorder Identification Test (10 items).

^f^TLFB: Timeline Follow Back for Alcohol Consumption.

^g^MAUQ: mobile health (mHealth) App Usability Questionnaire.

#### Baseline Measures

Upon successful registration, participants completed a baseline questionnaire to assess physical and mental health, health status, resource utilization, and sociodemographics.

#### Outcome Measures

The primary outcome was a change between baseline and day 84 follow-up in self-reported alcohol consumption, as measured by the 7-day TLFB. Participants were asked to report how many drinks they consumed over the past 7 days, as well as the type of drink consumed each day. Using standard unit measurements (see Multimedia Appendix 2 for an outline), the weekly alcohol consumption for baseline, day 28, day 84, and day 168 was determined by summing the number of units assigned to each drink.

The secondary outcome measure assessed changes in the AUDIT score from baseline to day 84 follow-up. The day 84 follow-up timepoint was selected to assess the short- to medium-term benefits of the intervention, although outcomes were also examined at day 168 to assess longer-term benefits. Changes in quality of life (eg, physical health, psychological health, social relationships, and environment) and cost-effectiveness will be analyzed in future papers.

At each follow-up, participants were first asked to complete the primary outcome assessment before continuing to complete the rest of the questionnaire. There were some cases where participants completed the primary outcome assessment but did not provide any data for the secondary outcome. Where no primary or secondary data points were provided, these were excluded for the specific analysis.

### Statistical Analysis

The statistical analysis plan was prospectively registered on the Open Science Framework [29]. Data were analyzed using Stata 16.1 MP (StataCorp).

Descriptive statistics were reported either as unweighted frequencies and percentages, the mean with the 95% CI, or the median with the IQR.

The primary and secondary outcomes were modeled using linear mixed effects models. Each outcome was tested in a separate model. Each model included up to 3 repeated outcome assessments, collected at days 28, 84, and 168. Repeated measures were clustered within individual participants, represented with a random intercept. The mixed model used all available information (ie, participants with at least 1 follow-up assessment were analyzed), leading to more precise estimates of the treatment effect.

We used multivariable binary logistic regression to assess whether baseline variables were associated with missingness in the primary outcome variable (1=missing primary outcome at day 84, 0=nonmissing). Each model included as covariates (1) time (measured as days since baseline), (2) a dummy variable to represent treatment allocation (0=control, 1=intervention), (3) a time × arm interaction term, (4) the baseline measurement of the outcome, (5) relevant covariates (age and gender), and (6) baseline variables associated with missingness (number of days off work due to alcohol consumption). Treatment effects were estimated as the difference between baseline and follow-up assessment (day 28, 84, or 168) and reported as the absolute alcohol unit difference between the arms.

Between-group effect sizes (Cohen *d*) were calculated by subtracting baseline total units consumed/AUDIT score from the day-of-assessment margin mean values (d=0.2, small effect; d=0.5, intermediate effect; and d=0.8, strong effect). The threshold for statistical significance reported in these analyses was *P*=.05.

The intention-to-treat analyses included all participants who completed at least 1 follow-up assessment (day 28, 84, or 168).

#### Sensitivity Analysis

We conducted a predefined sensitivity analysis in a subgroup of participants who had complete information for the primary outcome at day 84 (complete case analysis).

#### Process Evaluation

We examined process evaluation measures, used as a proxy for app usage. These were reported in 3 categories: (1) app utilization based on app analytics data provided by Google Analytics, (2) drinking analytics based on server interactions, and (3) notifications sent by the server. Where appropriate, these were reported either as the median with the IQR or as the mean with SD.

#### Usability

We examined usability of the *Drinks*:Ration app using the mobile health (mHealth) App Usability Questionnaire (MAUQ) [28] at day 28. Questionnaire responses were aggregated into (1) overall usability, (2) ease of use, (3) interface and satisfaction, and (4) usefulness. Results were summarized with means and SDs.

The study was also reported following the Template for Intervention Description and Replication [30] and the CONSORT (Consolidated Standards of Reporting Trials [31] and eHealth version [32]) checklist.

## Results

### Study Participation, Sample Characteristics, and Attrition

**Figure 2:**
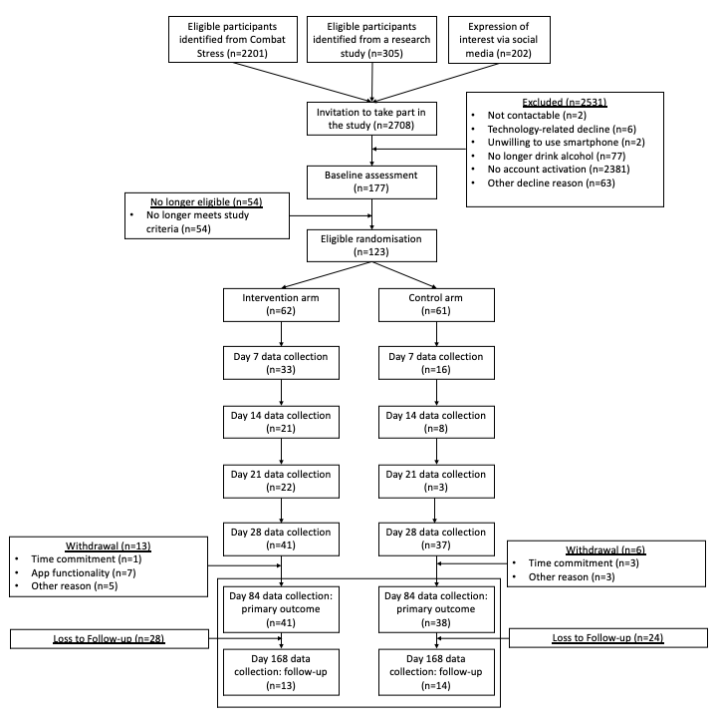
CONSORT diagram for recruitment into the RCT. CONSORT: Consolidated Standards of Reporting Trials; RCT: randomized controlled trial.

Between October 2020 and April 2021, 2708 individuals were invited to take part, of whom 2531 (93.5%) did not respond to the invite or declined to take part (n=150, 5.5%). In total, 177 (6.5%) participants were invited to complete a baseline assessment, of whom 54 (30.5%) were found to be ineligible based on study criteria (Figure 2). Therefore, a total of 123 (4.5%) participants completed the baseline assessment and were randomized into the study. Of these, 78 (63.4%) completed outcome assessments at day 28, 79 (64.2%) completed outcome assessments at day 84, and 27 (22.0%) completed outcome assessments at day 168. A total of 19 (15.4%) participants withdrew from the study by day 84. This included 7 (36.8%) participants who withdrew due to the limited functionality of the control version of the app.

Of the 123 participants, 62 (50.4%) participants were randomized to the intervention arm and 61 (49.6%) to the control arm. Baseline characteristics are shown in Table 2. The overall mean age was 47.6 years (95% CI 45.8-49.3), 117 (95.1%) participants were male, and 95 (77.2%) were married or in a long-term relationship. In addition, 87 (70.7%) had served in the army, and on average, the participants had served 14.4 years (95% CI 12.9-15.9) in the UK AF. The participants had a median AUDIT score of 16 (IQR 10-22) at baseline, and 66 (53.7%) were identified as having no probable PTSD. A total of 65 (52.9%) participants reported probable depression. Most participants entered the study with an Android device (n=67, 54.5%), and 79 (64.3%) participants completed the primary outcome assessment at day 84, with 76 (61.8%) completing the secondary outcome assessment.

**Table 2:** Descriptive statistics of eligible participants.

Characteristics		Total (N=123)	Control (N=61)	Intervention (N=62)
Age (years), mean (95% CI)		47.6 (45.8-49.3)	47.4 (44.9-50.0)	47.7 (45.3-50.1)
**Gender, n (%)**				
	Male	117 (95.1)	58 (95.1)	59 (95.2)
	Female	6 (4.9)	3 (4.9)	3 (4.8)
**Marital status, n (%)**				
	Married/in a relationship	95 (77.2)	48 (78.7)	47 (75.8)
	Single/separated	19 (15.5)	8 (13.1)	11 (17.7)
	Divorced/widowed	9 (7.3)	5 (8.2)	4 (6.5)
**Military branch, n (%)**				
	Royal Navy/Royal Marines	14 (11.4)	6 (9.8)	8 (12.9)
	Army	87 (70.7)	44 (73.2)	43 (69.6)
	Royal Air Force	16 (13.1)	8 (13.1)	8 (13.1)
	Other^a^	6 (4.9)	3 (4.9)	3 (4.9)
Length of military service in years, mean (95% CI)		14.4 (12.9-15.9)	15 (12.9-17.1)	13.8 (11.5-16.1)
Probable PTSD^b^, n (%)		57 (46.3)	26 (42.6)	31 (50.0)
Probable depression, n (%)		65 (52.9)	30 (49.2)	35 (56.5)
Probable anxiety, n (%)		61 (49.6)	32 (52.5)	29 (46.8)
AUDIT^c^ score, median (IQR)		16 (10-22)	14 (8-23)	16 (12-21)
Baseline unit weekly alcohol consumption^d^, median (IQR)		44 (25-70)	43 (25-62)	47 (26-73)
**Device type, n (%)**				
	iOS	56 (45.5)	32 (52.5)	24 (38.7)
	Android	67 (54.5)	29 (47.5)	38 (61.3)
Withdrawn by day 84, n (%)		19 (15.5)	13 (21.3)	6 (9.7)
**Completed primary outcome assessment, n (%)**				
	Day 28	78 (63.4)	37 (60.7)	41 (66.1)
	Day 84	79 (64.3)	38 (62.3)	41 (66.1)
	Day 168	27 (22.0)	14 (23.0)	13 (21.0)
**Completed secondary outcome assessment, n (%)**				
	Day 28	73 (59.4)	34 (55.7)	39 (62.9)
	Day 84	76 (61.8)	37 (60.7)	39 (62.9)
	Day 168	27 (22.0)	14 (23.0)	13 (21.0)

^a^Service branch not reported in medical records.

^b^PTSD: posttraumatic stress disorder.

^c^AUDIT: Alcohol Use Disorder Identification Test (10 items).

^d^Recorded using the Timeline Follow Back for Alcohol Consumption (TLFB).

### Primary and Secondary Outcome Analysis

For the primary outcome of the TLFB (units of alcohol per week) at day 84 (Table 3), we found that participants in the intervention arm had significantly larger reductions in self-reported alcohol unit consumption from baseline (marginal unit mean 56.3, 95% CI 50.6-62.0) to day 84 (marginal unit mean 28.1, 95% CI 21.1-35.1) compared with those in the control arm (marginal unit mean from 54.0, 95% CI 48.2-59.8, to 43.5, 95% CI 36.3-50.8; interaction *P*=.01). The effect size for the difference between the intervention and control arms in the mean change of units between baseline and day 84 was Cohen *d*=0.35, which is consistent with a moderate effect size.

Overall, we found that between baseline and day 84, weekly alcohol consumption reduced by –10.5 (95% CI –19.5 to –1.5) units in the control arm and –28.2 (95% CI –36.9 to –19.5) units in the intervention arm (*P*-value for the difference between arms at day 84=.003) at the primary outcome measure. The difference in unit marginal means was –15.4 (95% CI –25.5 to –5.4) units of alcohol in favor of the intervention arm.

**Figure 3:**
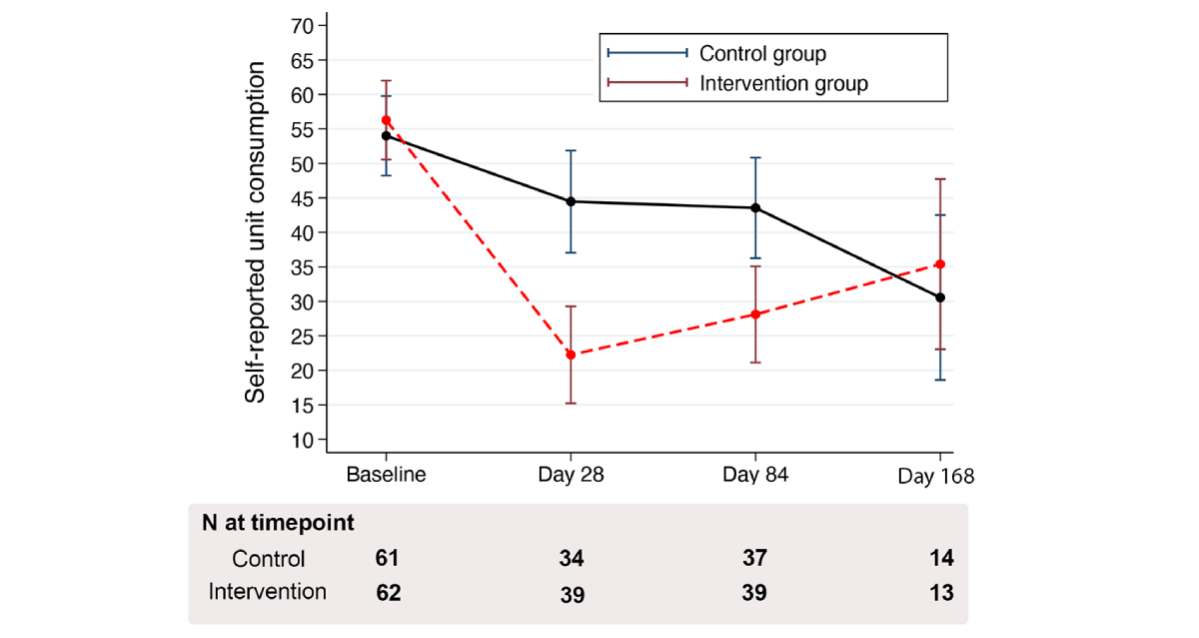
Trajectory for self-reported alcohol unit consumption per week as estimated from the mixed model.

There was evidence of a strong effect between the 2 arms by day 28 (Cohen *d*=0.50) but no evidence of a difference between the 2 arms by day 168 (Cohen *d*=0.11) for self-reported alcohol consumption (Figure 3).

For the secondary outcome change in the AUDIT score by day 84, we again found that participants in the intervention arm had significantly larger reductions in the AUDIT score from baseline (score marginal mean 16.3, 95% CI 15.0-17.5) to day 84 (score marginal mean 10.1, 95% CI 8.5-11.8) compared with those in the control arm (score marginal mean from 16.0, 95% CI 14.6-17.3, to 14.1, 95% CI 12.4-15.7; interaction *P*=.003). The difference was –3.9 (95% CI –6.2 to –1.6) points on the AUDIT score in favor of the intervention arm. The effect size for the difference between the intervention and control arms in the mean change in the AUDIT score between baseline and day 84 was Cohen *d*=0.48.

There was evidence of a strong effect between the 2 arms by day 28 (Cohen *d*=0.53) but no evidence of an effect between the 2 arms by day 168 (Cohen *d*=0.06) for the AUDIT score (Multimedia Appendix 3).

Sensitivity analyses of primary and secondary outcomes using complete case analysis produced the same patterns as those identified in the main analysis (Multimedia Appendix 4).

**Table 3:** Estimated mean change between each measure, timepoint, and arm. The difference in the rate of change between each arm compared with baseline is reported alongside the Cohen *d* statistic^a^.

Study arm		Estimated marginal mean (95% CI)					Evidence for a difference in rate of change between arms, interaction *P* value							Cohen *d*				
		Baseline	Day 28	Day 84	Day 168	Baseline-day 28			Baseline-day 84 (primary outcome)		Baseline-day 168 (secondary outcome)		Baseline-day 28		Baseline-day 84 (primary outcome)		Baseline- day 168 (secondary outcome)	
**Self-reported units consumed over the previous week**								<.001		.01		.80		0.50		0.35		0.11
	Control	54.0 (48.2-59.8)	44.5 (37.1-51.9)	43.5 (36.3-50.8)	30.6 (18.6-42.5)	N/A^b^			N/A		N/A		N/A		N/A		N/A	
	Intervention	56.3 (50.6-62.0)	22.2 (15.2-29.3)	28.1 (21.1-35.1)	35.4 (23.1-47.7)	N/A			N/A		N/A		N/A		N/A		N/A	
**AUDIT^c^ 10 score**								.001		.003		.68		0.53		0.48		0.07
	Control	16.0 (14.6-17.3)	16.5 (14.7-18.2)	14.1 (12.4-15.7)	13.2 (10.6-15.9)	N/A			N/A		N/A		N/A		N/A		N/A	
	Intervention	16.3 (15.0-17.5)	12.1 (10.5-13.7)	10.1 (8.5-11.8)	12.7 (9.9-15.4)	N/A			N/A		N/A		N/A		N/A		N/A	

^a^Derived from a model that was adjusted for age, gender, number of days off work due to alcohol consumption, and outcome measure.

^b^N/A: not appliable.

^c^AUDIT: Alcohol Use Disorder Identification Test (10 items).

### Process Evaluation

Over the entire study period (168 days), participants in the control arm used the app for a median of 1 week (IQR 1-2), initialized the app a median of 3 times (IQR 2-9), and had a median session duration of 60.9 seconds (IQR 35.7-75.6). Participants in the intervention arm used the app for a median of 3.5 weeks (IQR 2-6), initialized the app a median of 13.5 times (IQR 4-27), and had a median session duration of 43.8 seconds (IQR 32.3-67.9); see Table 4.

**Table 4:** Engagement and app interactions over the study period per participant stratified by arm.

Interactions		Control, median (IQR)	Intervention, median (IQR)
**Engagement measures**			
	Initializations	3 (2-9)	13.5 (4-27)
	Session count	24 (16-45)	54 (27-150)
	Session duration	60.9 (35.7-75.6)	43.8 (32.3-67.9)
	Server interactions	7 (5-8)	13 (8-19)
**App-recorded interactions**			
	Drinking days	N/A^a^	7 (4-11)
	Drink-free days	N/A	3.5 (2-7)
	Units consumed per drinking day	N/A	12.8 (4.4-16.5)
**Notifications**			
	Push notifications	1 (1-1)	18 (9-19)
	SMS^b^ text messages	2 (0-2)	12 (10-14)
Weeks active		1 (1-2)	3.5 (2-6)

^a^N/A: not applicable; participants in the control arm were not able to provide this information.

^b^SMS: short message service.

Participants in the intervention arm reported a median of 7 drinking days (IQR 4-11) during the first 28-day period, a median of 3.5 drink-free days (IQR 2-7), and a median of 12.8 units of alcohol per drinking day (IQR 4.4-16.5). A median of 18 push notifications (IQR 9-19) were sent to participants in the intervention arm, along with a median of 12 SMS text messages (IQR 10-14).

App use of participants in the intervention arm is shown in Table 5. Participants engaged with all modules of the app, but most of the app engagement was spent using the screening module (mean 201.0, SD 994.6) and the normative guidance module (mean 510.4, SD 1012.7).

**Table 5:** Intervention arm engagement with the *Drinks*:Ration app, stratified by page between baseline and day 168 based on app analytics data.

Page	Ever accessed^a^	Number of times accessed			Average time spent per session (seconds)
	n (%)	Mean (SD)	Median (IQR)	Median (IQR)	
Screening	62 (100)	4.9 (4.9)	2 (2-6)	27.7 (6.6-121.7)	
Normative Guidance	62 (100)	8.7 (9.9)	5.5 (3-10)	99.5 (34.5-459)	
Consent	62 (100)	3.3 (0.9)	3.5 (3-4)	53.8 (44.7-68.0)	
Dashboard	60 (96.8)	34.8 (60.5)	10.5 (4-42)	197.1 (85.0-360.8)	
Add Drinks	55 (88.7)	30.1 (53.0)	11 (3-38)	197.7 (47.8-408.5)	
Timeline Follow Back	52 (83.9)	3.1 (2.11)	2 (2-4)	1021.2 (700.1-1579.0)	
Drinks Diary Information	52 (83.9)	17.1 (23.3)	11 (2-22)	626.0 (117.2-1413.6)	
Drinks Diary	50 (80.7)	8.2 (13.1)	3 (1-8)	45.7 (16.5-198.8)	
View Goals	47 (75.8)	3.3 (0.7)	2 (1-4)	31.9 (3.1-63.4)	
User Account	40 (64.5)	3.0 (8.5)	1 (0-3)	17.3 (0-55.1)	

^a^During the study, Apple (developer of the iOS operating system) changed policies related to how developers could track and monitor usage of an app. This required specific user content, which could be modified outside the app. It is therefore not possible to ascertain whether a user did not give data because they were not using the app or whether they declined to share app usage statistics.

### Usability

The participants completed the MAUQ at day 28 (Table 6). They responded to a set of usability questions on a scale of 1-7, with a higher value indicating improved usability. Participants in the control arm reported a mean overall app usability score of 4.1 (SD 1.5), a mean ease-of-use score of 4.4 (SD 1.6), a mean interface and satisfaction score of 4.1 (SD 1.6), and a mean usefulness score of 3.6 (SD 1.7). These scores were lower than those of the intervention arm, which reported a mean overall app usability score of 5.9 (SD 1.1), a mean ease-of-use score of 5.9 (SD 1.2), a mean interface and satisfaction score of 5.9 (SD 1.1), and a mean usefulness score of 5.7 (SD 1.1).

**Table 6:** MAUQ^a^ results at day 28, stratified by arm.

Items	Control (N=35), mean (SD)	Intervention (N=38), mean (SD)
Ease-of-use	4.4 (1.6)	5.9 (1.2)
Interface and satisfaction	4.1 (1.6)	5.9 (1.1)
Usefulness	3.6 (1.7)	5.7 (1.3)
Overall	4.1 (1.5)	5.9 (1.1)

^a^MAUQ: mobile health (mHealth) App Usability Questionnaire.

### Adverse Events and Technical Issues

In total, 2 (1.6%) of 123 participants were identified as having a single adverse event of consuming more than 25 units of alcohol within 24 hours during the study period. Following our risk protocol [19], a signposting booklet to relevant charities was provided, as well as a call with the study clinical lead. After a clinical interview, both participants were allowed to continue in the study. Their treatment allocation was not disclosed to the participants. No other adverse events were identified. No technical issues occurred during the trial.

## Discussion

### Principal Findings

This study is 1 of the few RCTs to date to examine the efficacy of a fully automated 28-day brief alcohol intervention delivered via a mobile app in a help-seeking sample of UK veterans with at least hazardous alcohol consumption. Help-seeking veterans were consuming on average 55 units of alcohol per week at the outset of the study, well above the 14 units of alcohol per week recommended as the maximum by the UK Chief Medical Officer. At the primary outcome (day 84), the difference between the estimated marginal means for the intervention and control arms was 15.4 units of alcohol lower in the intervention arm than in the control arm. A similar pattern was also observed for the AUDIT score, where the difference between the estimated marginal mean between the arms was 4.0 points on the AUDIT scale, also lower than that of the control arm. Overall, the intervention arm achieved significantly better reductions in alcohol consumption and AUDIT score. These effects disappeared at day 168.

The findings of this RCT demonstrate the efficacy of an automated brief alcohol intervention with personalized messaging for those who consume alcohol at least at hazardous levels and have sought help for a mental health difficulty [15]. The findings also mirror those obtained in other studies [7,12]. In particular, the findings demonstrate the efficacy of *Drinks*:Ration within a group that has been shown to be at increased risk of dual diagnosis. The between-arm difference compares favorably to stand-alone self-help interventions [33], and the differences are greater than those typically found with face-to-face therapies and the most successful therapist-guided interventions [34].

This study found significant differences between the arms for the primary and secondary outcomes at the primary data collection point (day 84), and the differences were even larger at day 28 when the first assessment took place. At follow-up (day 168), only 22% of participants responded, which limited our ability to discern differences at the timepoint. An alternative explanation is that the effect of the intervention reduced over time, so the long-term benefits of the *Drinks*:Ration app may need reinforcing beyond the intervention month (ie, the first 28 days).

The results of this RCT should be placed in the context of the wider literature. A recent literature review exploring the effectiveness of alcohol reduction apps and the availability of evidenced-based apps on top commercial app stores identified 21 articles representing 19 unique mobile apps [35]. Of these, 7 (36.8%) apps were targeted at adolescent drinkers, and the remainder on the general population. No studies that targeted the AF were identified. The overall effectiveness of the included interventions was mixed, with standards of reporting making direct comparisons difficult (eg, AUDIT-C, binge-drinking days, alcohol unit consumption). *Drinks*:Ration results compare favorably to all included studies, in so far as we identified the largest reductions in alcohol consumption; however, our base starting point was significantly higher than the general population.

Although there are no reported data on waiting times between referral and treatment in the United Kingdom, National Health Service (NHS) Scotland national drug and alcohol treatment waiting times for alcohol treatment were reported to be around 3 weeks or less between January 2021 and March 2021 [36]. The delay between referral and treatment may be an opportunity to deploy the *Drinks*:Ration app to support help-seeking veterans while they wait for formal treatment. Help-seeking veterans misusing alcohol attend fewer mental health appointments [6], probably because many are prevented from receiving treatment for mood disorders and PTSD until they have reduced their excessive drinking. The use of the *Drinks*:Ration app to support reduction in alcohol consumption could enable more help-seeking veterans to access services. In addition to the *Drinks*:Ration app, the use of personalized messages sent via SMS and push notification may have contributed to improved performance of the intervention arm.

The efficacy of the app in relation to our primary outcome is encouraging, but there are some issues worth considering. The recruitment for this study was lower than anticipated. We expected that 30% of eligible veterans would enter the study, but though it was difficult to estimate the true total number of eligible individuals, the percentage who consented to participate was less than 10%. The second potential problem is that the effect seen at 84 days may need a reinforcement to encourage persistent changes in behavior over the long term. This could be achieved by enabling personalized messaging over the entire life course of app usage.

There were 2 adverse events (involving 2 participants), which were unlikely to be caused by our app and more likely to be caused by the ongoing alcohol consumption of the individuals involved. This highlights the challenges of monitoring adverse events in remote/automated interventions for which the implementation of the app needs constant monitoring while being used. Finally, it is important to consider how *Drinks*:Ration can be integrated into the treatment pathway to support veterans prior and during treatment, while also monitoring the degree to which its efficacy transfers to a clinical context.

### Limitations

Several limitations of this trial should be noted. First, as already acknowledged, the majority of those invited to participate in the study did not take part. It is not possible to ascertain why these individuals chose not to take part, but it may be due to digital fatigue because of the COVID-19 pandemic. Therefore, we consider that our study assessed the efficacy of the intervention in those willing to engage with the app rather than effectiveness in the target population. Second, participants self-identified their military and help-seeking status among those recruited through social media and the status was not verified. Third, we only used self-reported data provided via outcome assessments and did not use data collected via the drinks diary. This decision to use the TLFB for assessing our primary outcome was to ensure comparability with other studies and to also ensure the control arm did not complete the drinks diary. This resulted in duplicating participant input, which could have created user frustration and negatively impacted usability and participation. Finally, this RCT was conducted during the COVID-19 pandemic. This period resulted in meaningful behavioral changes to how UK military veterans consumed alcohol due to lockdown. In a recent study of UK veterans, they were found to be drinking less alcohol during the first phase of the pandemic, reducing their hazardous drinking from 49% to 28% [37]. This may have reduced the available population that consumes alcohol at a harmful-to-hazardous level.

### Conclusion

Our findings suggest that *Drinks*:Ration is efficacious in reducing alcohol consumption in help-seeking veterans and that wider uptake of *Drinks*:Ration in this population would be beneficial. However, strategies to increase use of the app and ensure that the gains in decreasing alcohol consumption persist over time need to be well thought out. This could be achieved by promoting app use and continuation of messaging and more personalized goal setting.

## Appendix

Multimedia Appendix 1 Messaging: push notifications and SMS texts. SME: short message service.

Multimedia Appendix 2 Standard units.

Multimedia Appendix 3 Trajectory for AUDIT outcome from the mixed models. AUDIT: Alcohol Use Disorder Identification Test.

Multimedia Appendix 4 Complete case analysis.

Multimedia Appendix 5 CONSORT-eHEALTH checklist (V 1.6.1).

## Abbreviations

AF: armed forces
AUDIT: Alcohol Use Disorder Identification Test
BCT: behavior change technique
InDEx: Information about Drinking for Ex-Serving personnel
MAUQ: mobile health (mHealth) App Usability Questionnaire
PTSD: posttraumatic stress disorder
RCT: randomized controlled trial
SMS: short message service
TLFB: Timeline Follow Back for Alcohol Consumption

## Data Availability

Due to the sensitive nature of the cohort, access to data is limited. Researchers may apply to access a pseudonymized data set. Requests to access study data are subject to submission of a research proposal to the corresponding author. All requests must be made in accordance with the UK Policy Framework for Health and Social Care research. Where the applicant is outside of King’s College London, a data-sharing agreement is required.
