# Supplementary material for: Evaluating the Efficacy of the Drinks:Ration Mobile App to Reduce Alcohol Consumption in a Help-Seeking Military Veteran Population: Randomized Controlled Trial
Source: JMIR Mhealth Uhealth. 2022 Jun 20;10(6):e38991. doi: 10.2196/38991 (PMC9254042; doi:10.2196/38991)
Supplement: Multimedia Appendix 1 [file mhealth_v10i6e38991_app1.docx]

**Appendix A: Messaging - push notifications and SMS messaging**

In addition to the *Drinks*:Ration app, participants received personalised push notifications and SMS messaging which provided prompts to use of the drinks diary, completion of questionnaires, suggest alternative behaviours, provides guidance on goals and to promote healthy lifestyle. A bank of personalised messages (both for SMS text message and push notifications) informed by 180 tailored messages developed during the feasibility trial were used. These are informed by the Health Action Process Approach framework and targeted towards specific BCTs.

*Drinks*:Ration uses baseline and contiguous measurements (day 7/14/21) to inform the type of message a participant receives to provide an individual participant-centric approach. Baseline measurements are used to identify suitable messages and as a participant engages with *Drinks*:Ration, continuous measurements including questionnaires (baseline and weekly questionnaires) and the drinks diary are used to reflect current behaviour and attitude. The messages cover a wide range of topics to target beliefs and motivations with the primary aim of increasing the participant’s awareness of their drinking habits and behaviours. The messages are divided into 3 categories:

(1) tailored: personalised to drinking habits, baseline, and weekly questionnaires;

(2) tailored and triggered: tailored to baseline and contiguous measurements and a specific event occurring; and

(3) targeted (generic): sent on specific days to highlight inactivity, reminder to complete a questionnaire, or to alter participants to a new feature.

A participant could receive at most 20 messages over the primary 28-day period, with a maximum of two in a single day. The system automatically decided when a message should be sent and the mode of delivery (push notification or SMS messaging). A list of targeted (generic) which were sent to participants is defined in Table 7; except the messages listed in this table, participants in the control arm will received no other messaging. Participants in both arms received a generic message designed to promote retention of the *Drinks*:Ration app to allow for follow-up data collection. This will be sent monthly.

| **Day** | **Message Content** | **Arm** |
| --- | --- | --- |
| 0 | Title: Welcome to *Drinks*:Ration.  Message: Your signup is complete. Remember to log last week’s alcohol consumption… | I |
| 0 | Title: Welcome to *Drinks*:Ration.  Message: Your signup is now complete. | C |
| 2 | Title: Time to set a goal?  Message: Why not set a goal to reduce the amount you drink? You can start now by clicking on the ‘goals’ tab in the app… | I |
| 7 | Title: Drinking Advice  Message: Remember to open *Drinks*:Ration for advice on how to make your alcohol consumption | C |
| 13 | Title: Remember the diary  Message: Completing the drinks diary each day allows you to see how well you are doing! | I |
| 14 | Title: Let us know…  Message: Remember to open the app to tell us how you’re doing | I/C |
| 18 | Title: Remember to monitor  Message: Monitoring what you drink is proven to help you cut down - why not start fresh this week? | I |
| 21 | Title: Be aware!  Message: It is good to be aware of what you drink. Monitoring your alcohol consumption really can help your health! | I/C |
| 23 | Title: Think…  Message: Using Drinks:Ration may have changed some of the drinks you usually have. Try and think how you can keep it up! | I |
| 28 | Title: Let us know…  Message: Please let us know how you’re getting on! | I/C |
| 56 | Title: Keep checking in…  Message: Please remember to open the Drinks:Ration app | I/C |
| 84 | Title: Let us know…  Message: Please let us know how you’re getting on! | I/C |
| 112/140 | Title: Keep checking in…  Message: Please remember to open the Drinks:Ration app | I/C |
| 168 | Title: Let us know…  Message: Please let us know how you’re getting on! | I/C |

I: Intervention Arm; C: Control Arm.
