# Supplementary material for: Evaluating the Efficacy of the Drinks:Ration Mobile App to Reduce Alcohol Consumption in a Help-Seeking Military Veteran Population: Randomized Controlled Trial
Source: JMIR Mhealth Uhealth. 2022 Jun 20;10(6):e38991. doi: 10.2196/38991 (PMC9254042; doi:10.2196/38991)
Supplement: Multimedia Appendix 3 [file mhealth_v10i6e38991_app3.docx]

**Appendix C: Trajectory for AUDIT outcome from the mixed models**
