# Supplementary material for: Evaluating the Efficacy of the Drinks:Ration Mobile App to Reduce Alcohol Consumption in a Help-Seeking Military Veteran Population: Randomized Controlled Trial
Source: JMIR Mhealth Uhealth. 2022 Jun 20;10(6):e38991. doi: 10.2196/38991 (PMC9254042; doi:10.2196/38991)
Supplement: Multimedia Appendix 4 [file mhealth_v10i6e38991_app4.docx]

**Appendix D: Complete case analysis**

|  | Baseline | Day 28 | Day 84 | Day 168 | Baseline  to  Day 28 | Baseline  to  Day 84  Primary Outcome | Baseline  to  Day 168  Secondary Outcome | Baseline  to  Day 28 | Baseline  to  Day 84  Primary Outcome | Baseline  to  Day 168  Secondary Outcome |
| --- | --- | --- | --- | --- | --- | --- | --- | --- | --- | --- |
|  | Estimated Marginal Mean  (95% CI) | Estimated Marginal Mean  (95% CI) | Estimated Marginal Mean  (95% CI) | Estimated Marginal Mean  (95% CI) | Evidence for a difference in rate of change between arms  Interaction *p* value | Evidence for a difference in rate of change between arms  Interaction *p* value | Evidence for a difference in rate of change between arms  Interaction *p* value | Cohen’s D | Cohen’s D | Cohen’s D |
| **Self-reported units consumed over the previous week** | | | | | | | | | | |
| Control (n=38) | 52.57  (45.23 to 59.91) | 43.21  (34.80 to 51.62) | 42.55  (35.22 to 49.89) | 28.79  (16.69 to 40.89) | .001 | .015 | .696 | 0.55 | 0.35 | 0.15 |
| Intervention (n=41) | 54.61  (47.57 to 61.64) | 18.90  (11.18 to 26.62) | 27.17  (20.13 to 32.21) | 34.85  (21.88 to 47.81) |  |  |  |  |  |  |
| **AUDIT 10 score** | | | | | | | | | | |
| Control (n=37) | 14.77  (13.14 to 16.40) | 14.71  (12.74 to 16.69) | 13.28  (11.66 to 14.91) | 12.47  (9.86 to 15.08) | .011 | .002 | .842 | 0.39 | 0.44 | 0.06 |
| Intervention (n=39) | 15.76  (14.17 to 17.34) | 11.51  (9.77 to 13.24) | 9.70  (8.12 to 11.29) | 13.03  (10.25 to 15.81) |  |  |  |  |  |  |

Note: Derived from model which was adjusted for age, sex, number of days off work due to alcohol consumption and outcome measure.
